# Response to commentary on a trial comparing krill oil versus fish oil

**DOI:** 10.1186/1476-511X-13-17

**Published:** 2014-01-22

**Authors:** Vanu R Ramprasath, Inbal Eyal, Sigalit Zchut, Peter JH Jones

**Affiliations:** 1 Richardson Centre for Functional Foods and Nutraceuticals, University of Manitoba, 196 Innovation Drive, Winnipeg MB R3T 2N2, Canada; 2 Department of Human Nutritional Sciences, University of Manitoba, Winnipeg, Canada; 3 Enzymotec Ltd., P.O.B 6 Migdal HaEmeq, Israel

**Keywords:** Krill oil, Fish oil, Fatty acids

## Abstract

Nichols et al. (*Lipids Health Dis***13:**2, 2014) raised concern about the higher n-6 concentration in fish oil used in our recent study which is different from typical commercial fish oils (Ramprasath et al. *Lipids Health Dis***12:**178, 2013). The aim of our study was to compare the effect of consumption of similar amount of n-3 PUFA from krill and fish oil with placebo on plasma and RBC fatty acids. As the concentration of n-3 PUFA in the fish oil utilised was higher than that in krill oil, we deemed it important to keep consistent the concentration of n-3 PUFA and volumes to be administered to participants between krill versus fish oils. As such, the fish oil used in the study was diluted with corn oil. Although the n-6 PUFA concentration in fish oil was higher compared to traditionally used fish oil, consumption of the fish oil used in our study actually reduced the total n-6 PUFA in plasma and RBC to a similar extent as did krill oil. Overall, our conclusion was that the increases in plasma and RBC concentrations of EPA and DHA along with improvement in the omega-3 index observed with consumption of krill oil compared with fish oil are due to differences in absorption and bioavailability based on the structural difference of the two oils rather than their n-6 PUFA content.

## Background

Recently, Ramprasath et al. [1] compared effects of consumption of 600 mg/day of n-3 PUFA from krill or fish oil versus placebo corn oil by healthy individuals for 4 weeks separated by 8 week wash out periods using a double blind randomised cross over study design. Results showed increased plasma and RBC n-3 PUFA concentrations after consumption of n-3PUFA from the krill compared to fish oil. Nichols et al. [2] raised an issue regarding reported fatty acid profile of fish oil used in that it possessed high concentrations of n-6 PUFA. However this assessment did not take into consideration that the n-3 PUFA concentrations of krill and fish oils are not similar and hence the concentration of n-3 PUFA between the two oils needed to be adjusted for consistency.

## Discussion

Our primary objective was to compare effects of consumption of same amount of n-3 fatty acids from krill or fish oil. When designing a double blinded placebo controlled randomised cross over trial, it was felt that the amounts of treatment products as well as the bioactives of interest be maintained consistent across different interventions. However, the n-3 PUFA content of the krill oil fell below that of fish oil. In order to match the concentrations of n-3 PUFA and volumes between krill and fish oil, the fish oil was diluted with the placebo, corn oil at a ratio of 1.3:1.0. We agree that we could have included the information about dilution of fish oil in the original manuscript itself. Addition of corn oil, to fish oil resulted in higher total n-6 PUFA concentration in fish oil. We agree that one of the limitations of the study is not controlling the n-6 fatty acid content between the different oils, as we mention in the discussion and study limitations sections of the manuscript [1]. We concede that some influence due to the difference in n-6 PUFA concentration might have occurred. However, we chose to control only the n-3 PUFA content which is the most important component of interest in the two oils.

Furthermore, dietary n-6 PUFA consumption by the participants was not controlled during the study. The reason to control the n-3 and not the n-6 PUFA was based on the knowledge we have regarding the typical Western diet which contains high amounts of n-6 PUFA, up to 25 times more than n-3 PUFA [3]. Consumption level of linoleic acid by an American adult approaches 17 grams per day on average for males and 13 grams per day for females [4]. Thus, variations in amounts of n-6 PUFA in the diet are foreseeably greater than the difference in their amounts between the different treatment oils. It is relevant to point out that fish oil reduced the total n-6 PUFA in plasma as well as RBC to similar extent as krill oil despite having a higher n-6 PUFA content in the fish oil [1]. Accordingly, we suggest that increased plasma and RBC n-3 PUFA concentrations with krill oil consumption compared with fish oil might be due to differences in absorption and bioavailability based on the structural difference of the two oils rather than their n-6 PUFA content.

Plasma linoleic acid levels were significantly decreased after both krill and fish oil consumption compared with placebo. However, no significant difference in the plasma end point concentration was observed for linoleic acid between krill and fish oils. Furthermore, no changes in linoleic acid concentrations within RBCs were seen with any of the oil treatments [1]. Hence, although a difference existed in the n-6 PUFA concentration and ratio between n-6 and n-3 PUFA between krill and fish oils, their effects on the plasma and RBC n-6 PUFA and linoleic acids were shown to be almost identical. It has also been previously shown that a half dose of krill oil has a similar effect as a single dose of fish oil on increasing plasma n-3 PUFA concentrations [5].

Another comment Nichols et al. raised was regarding variations in the levels of n-3 PUFA in the fish and krill oil supplements (Table 1 in the original manuscript) [2]. Nichols and colleagues might have made an erroneous assumption when they calculated the percent of fatty acids as percent of the entire oil. As indicated (Table 6 in the original manuscript) [1], the amount of fatty acids is given as percent of total fatty acids, not of the total oil. Thus, the amount of n-3 PUFA consumed from both krill and fish oil was 600 mg per day in our study [1].

## Conclusion

In summary, although, the n-6 PUFA concentration in fish oil used in the study was higher than that of the krill oil, the effect of consumption of these two oils resulted in similar perturbations on the plasma n-6 PUFA concentrations. As such, the greater increase in concentrations of EPA and DHA in plasma and RBC along with improvement in the omega-3 index following krill oil compared with fish oil consumption is due to increased bioavailability of n-3 PUFA from krill oil than fish oil.

## Abbreviations

DHA: Docosahexaenoic acid; EPA: Eicosapentaenoic acid; PUFA: Polyunsaturated fatty acids; TG: Triglycerides.

## Competing interests

VRR and PJHJ declare no conflict of interest; IE and SZ are employees of Enzymotec Ltd.

## Authors’ contributions

VRR and PJHJ conceived the content of manuscript and participated in drafting the manuscript. All authors read and approved the final manuscript.
